# A Subset of Exoribonucleases Serve as Degradative Enzymes for pGpG in c-di-GMP Signaling

**DOI:** 10.1128/JB.00300-18

**Published:** 2018-11-26

**Authors:** Mona W. Orr, Cordelia A. Weiss, Geoffrey B. Severin, Husan Turdiev, Soo-Kyoung Kim, Asan Turdiev, Kuanqing Liu, Benjamin P. Tu, Christopher M. Waters, Wade C. Winkler, Vincent T. Lee

**Affiliations:** a Department of Cell Biology and Molecular Genetics, University of Maryland, College Park, Maryland, USA; b Department of Biochemistry & Molecular Biology, Michigan State University, East Lansing, Michigan, USA; c Microbiology & Molecular Genetics, Michigan State University, East Lansing, Michigan, USA; d Department of Biochemistry, University of Texas Southwestern Medical Center, Dallas, Texas, USA; Geisel School of Medicine at Dartmouth

**Keywords:** cyclic-di-GMP signaling, nanoRNase, RNA degradation, dinucleotide hydrolysis, pApA, pGpG

## Abstract

Bis-(3′-5′)-cyclic dimeric GMP (c-di-GMP) is a bacterial second messenger that regulates processes, such as biofilm formation and virulence. During degradation, c-di-GMP is first linearized to 5′-phosphoguanylyl-(3′,5′)-guanosine (pGpG) and subsequently hydrolyzed to two GMPs by a previously unknown enzyme, which was recently identified in Pseudomonas aeruginosa as the 3′-to-5′ exoribonuclease oligoribonuclease (Orn). Mutants of *orn* accumulated pGpG, which inhibited the linearization of c-di-GMP. This product inhibition led to elevated c-di-GMP levels, resulting in increased aggregate and biofilm formation. Thus, the hydrolysis of pGpG is crucial to the maintenance of c-di-GMP homeostasis. How species that utilize c-di-GMP signaling but lack an *orn* ortholog hydrolyze pGpG remains unknown. Because Orn is an exoribonuclease, we asked whether pGpG hydrolysis can be carried out by genes that encode protein domains found in exoribonucleases. From a screen of these genes from Vibrio cholerae and Bacillus anthracis, we found that only enzymes known to cleave oligoribonucleotides (*orn* and *nrnA*) rescued the P. aeruginosa Δ*orn* mutant phenotypes to the wild type. Thus, we tested additional RNases with demonstrated activity against short oligoribonucleotides. These experiments show that only exoribonucleases previously reported to degrade short RNAs (*nrnA*, *nrnB*, *nrnC*, and *orn*) can also hydrolyze pGpG. A B. subtilis *nrnA nrnB* mutant had elevated c-di-GMP, suggesting that these two genes serve as the primary enzymes to degrade pGpG. These results indicate that the requirement for pGpG hydrolysis to complete c-di-GMP signaling is conserved across species. The final steps of RNA turnover and c-di-GMP turnover appear to converge at a subset of RNases specific for short oligoribonucleotides.

**IMPORTANCE** The bacterial bis-(3′-5′)-cyclic dimeric GMP (c-di-GMP) signaling molecule regulates complex processes, such as biofilm formation. c-di-GMP is degraded in two-steps, linearization into pGpG and subsequent cleavage to two GMPs. The 3′-to-5′ exonuclease oligoribonuclease (Orn) serves as the enzyme that degrades pGpG in Pseudomonas aeruginosa. Many phyla contain species that utilize c-di-GMP signaling but lack an Orn homolog, and the protein that functions to degrade pGpG remains uncharacterized. Here, systematic screening of genes encoding proteins containing domains found in exoribonucleases revealed a subset of genes encoded within the genomes of Bacillus anthracis and Vibrio cholerae that degrade pGpG to GMP and are functionally analogous to Orn. Feedback inhibition by pGpG is a conserved process, as strains lacking these genes accumulate c-di-GMP.

## Abstract (precis)

The bacterial bis-(3′-5′)-cyclic dimeric GMP (c-di-GMP) signaling molecule regulates complex processes, such as biofilm formation. c-di-GMP is degraded in two-steps, linearization into pGpG and subsequent cleavage to two GMPs. The 3′-to-5′ exonuclease oligoribonuclease (Orn) serves as the enzyme that degrades pGpG in Pseudomonas aeruginosa. Many phyla contain species that utilize c-di-GMP signaling but lack an Orn homolog, and the protein that functions to degrade pGpG remains uncharacterized. Here, systematic screening of genes encoding proteins containing domains found in exoribonucleases revealed a subset of genes encoded within the genomes of Bacillus anthracis and Vibrio cholerae that degrade pGpG to GMP and are functionally analogous to Orn. Feedback inhibition by pGpG is a conserved process, as strains lacking these genes accumulate c-di-GMP.

## INTRODUCTION

The Benziman lab described bis-(3′-5′)-cyclic dimeric GMP (c-di-GMP) in 1987 as an allosteric activator of cellulose synthase in Acetobacter xylinus (since renamed Komagataeibacter xylinus) (1). c-di-GMP is utilized by many bacterial species to govern behaviors, such as biofilm formation, motility, virulence, development, and cell cycle progression, making c-di-GMP a crucial regulator of bacterial lifestyle transitions. In general, high levels of c-di-GMP promote a sessile biofilm-forming lifestyle, while low levels of c-di-GMP promote a motile planktonic lifestyle (see reference 2 for a comprehensive review of c-di-GMP signaling).

In their initial report, the Benziman lab demonstrated that c-di-GMP is synthesized from two GTP molecules by enzymes with diguanylate cyclase (DGC) activity. c-di-GMP is degraded to two GMP molecules via a two-step process. First, it is hydrolyzed into linear 5′-phosphoguanylyl-(3′,5′)-guanosine (pGpG) by enzymes the authors referred to as phosphodiesterase A. This linearization process can be inhibited by Ca^2+^ ions (1), while the subsequent hydrolysis of pGpG to two GMPs is not inhibited by Ca^2+^, which the authors interpreted as evidence for a second distinct enzyme which they termed phosphodiesterase B (1). Numerous follow-up experimental and bioinformatics studies revealed the motifs and domains for DGC activity (GGDEF domains) (3–5) and c-di-GMP linearization activity (EAL [4, 6, 7] and HD-GYP [8] domains), yet the identity of the enzyme responsible for pGpG cleavage remained unknown. While EAL domain and HD-GYP proteins have been shown to degrade pGpG *in vitro*, their contribution to pGpG turnover in bacterial cells remains under investigation. Recently, two publications identified Orn as the primary phosphodiesterase B (PDE-B) in Pseudomonas aeruginosa (9, 10). Using cell lysates, we showed that ^32^P-labeled pGpG is turned over at a much lower rate in the PA14 Δ*orn* mutant than in the wild type (9). The Δ*orn* mutant likely continued to express EAL and HD-GYP domain proteins, but their contribution toward pGpG turnover was less than 5% of that of Orn, indicating that Orn is the primary enzyme responsible for pGpG hydrolysis *in vivo*.

While c-di-GMP signaling is used across the bacterial domain, homologs of *orn* are restricted to Betaproteobacteria, Deltaproteobacteria, Gammaproteobacteria, and Actinobacteria (9). For bacterial phyla that utilize c-di-GMP signaling but lack *orn* homologs, these organisms must encode another group of enzymes that fulfill the role of Orn in pGpG cleavage. Orn is a 3′-to-5′ exoribonuclease that is the major enzyme responsible for degrading short oligoribonucleotides in Escherichia coli. Orn was first isolated from E. coli in the 1970s and shown to degrade short (5-mer and shorter) poly(A) oligonucleotides *in vitro* (11, 12). The *orn* gene is essential in E. coli. To determine the function of Orn *in vivo*, a temperature-dependent mutant was generated by introducing a chromosomal interruption in the *orn* locus while supplying *orn* on a temperature-sensitive plasmid (13). Upon growth of this temperature-dependent E. coli *orn* mutant under nonpermissive conditions, the strain accumulated oligoribonucleotides that are 2 to 5 nucleotides long (13). In bacterial species that do not contain an *orn* homolog, other RNases were later identified to degrade oligoribonucleotides by screening for genes that rescue growth of the E. coli *orn* mutant. Genes that rescued the *orn* mutant included those coding for NrnA and NrnB, which are widely found in Firmicutes (14, 15), and for NrnC, which is widely found in Alphaproteobacteria (16). However, direct evidence of a role in degradation of short RNA *in vivo* was lacking. In addition, two RNases, YhaM and RNase J1, from B. subtilis also partially rescued the E. coli conditional *orn* deletion mutant (15). *In vitro*, the 3′-to-5′ exoribonuclease YhaM (17) can degrade 5-mer oligonucleotide RNA but was able to degrade oligonucleotide DNA at a higher rate, suggesting that DNA could be a preferred substrate (15). The 5′-to-3′ exoribonuclease RNase J1 (18) had low activity *in vitro* against 5-mer cytosine and adenine (15). These reports suggest that other RNases may degrade pGpG to terminate c-di-GMP signaling in species that lack *orn*. Currently, these candidates have not been experimentally tested for hydrolysis of pGpG and their effects on c-di-GMP signaling. We thus used a similar complementation approach to assay the effect of RNases on pGpG turnover.

P. aeruginosa Δ*orn* mutants are viable but have increased levels of cytosolic c-di-GMP due to pGpG feedback inhibition, resulting in elevated c-di-GMP-regulated processes, such as biofilm formation (19, 20). We hypothesized that genes encoding domains found in known RNA exonucleases could cleave pGpG in species that do not encode *orn* and should be able to restore the behavior of the P. aeruginosa Δ*orn* strain to that of the wild type. Thus, we identified genes that contained domains found in RNA exoribonucleases from B. anthracis, an organism that lacks *orn*, and Vibrio cholerae, another species that contains *orn* and is well known to utilize c-di-GMP signaling and thus may encode additional proteins for pGpG turnover. These genes were tested for their ability to degrade pGpG through complementation of the P. aeruginosa Δ*orn* strain. Of the genes tested, only the known “nanoRNases,” including Orn, NrnA, NrnB, and NrnC, could reduce aggregation of the P. aeruginosa Δ*orn* strain to wild-type levels. Cells that express NrnA, NrnB, and NrnC reduced levels of pGpG and c-di-GMP found in the P. aeruginosa Δ*orn* strain. Purified recombinant NrnA, NrnB, and NrnC proteins were able to cleave pGpG in a manner similar to Orn. Bacillus subtilis lacking both *nrnA* and *nrnB* accumulated c-di-GMP. These results demonstrate that a specific subset of RNases act to hydrolyze pGpG, indicating that RNases serve as the final processing enzyme to terminate c-di-GMP signaling across bacteria.

## RESULTS

### A screen identifies exoribonucleases that rescue cell aggregation and biofilm formation in P. aeruginosa PA14 Δ*orn*.

A bioinformatic approach was used to identify candidate exoribonucleases for screening to identify additional enzymes responsible for turning over pGpG. Previously reported exoribonucleases in E. coli and B. subtilis include oligoribonuclease, RNase B, RNase BN, RNase D, RNase J, RNase PH, RNase R, RNase T, PNPase, YhaM, and Nrn proteins (21–24). These proteins were used as a starting point for bioinformatic identification of putative exoribonucleases based on Pfam domains (see Table S1 in the supplemental material). The Pfam HMM model obtained from the Pfam database version 31 (March 2017) was searched against the complete proteomes of B. anthracis strain Ames and V. cholerae serotype O1 using the HMMER 3.1b2 hmmsearch command (25); this resulted in a list of 51 unique protein sequences with a significant E value, as reported by HMMER (see Table S1) (26). Of these 51 sequences, 50 genes were obtained from B. anthracis and V. cholerae (27) Gateway clone set libraries (*polC* [*BA3955*] was not available) and introduced into a replicative plasmid in P. aeruginosa.

**FIG 1:**
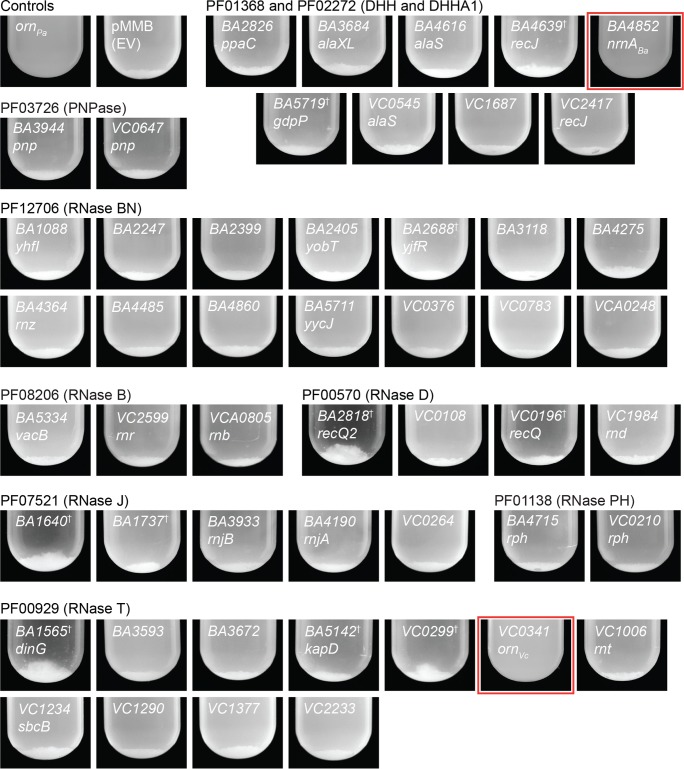
A subset of genes with RNase domains reduce aggregation by PA14 Δ*orn*. Photograph of overnight cultures of PA14 Δ*orn* with empty vector (EV) and complementation vectors expressing the indicated genes. Genes are grouped by RNase domain. Strains were growing with shaking and induction overnight, allowed to sediment for 30 min by gravity, and photographed. Daggers indicate strains grown and induced at 30°C, while the remaining strains were grown and induced at 37°C. Red boxes indicate genes that prevented autoaggregation.

The PA14 Δ*orn* strain has elevated levels of pGpG and c-di-GMP, resulting in increased autoaggregation (Fig. 1) (9). The ability of each of the 50 genes to cleave pGpG was tested by transcomplementation of the PA14 Δ*orn* strain to reduce autoaggregation. Expression of the PA14 *orn* (*orn_Pa_*) complemented the PA14 Δ*orn* mutant and prevented aggregate formation, whereas the vector control aggregated. The expression of genes encoding RNase B, RNase BN, RNase D, RNase J, RNase PH, or PNPase domains in the Δ*orn* mutant did not prevent aggregation, indicating that they do not hydrolyze pGpG (Fig. 1). Of the genes encoding the RNase T domain, only *VC0341* (*orn_Vc_*) from V. cholerae was able to reverse aggregation (Fig. 1). For genes encoding DHH or DHHA1 domains, only *BA4852* (*nrnA_Ba_*) from B. anthracis prevented aggregation.

**FIG 2:**
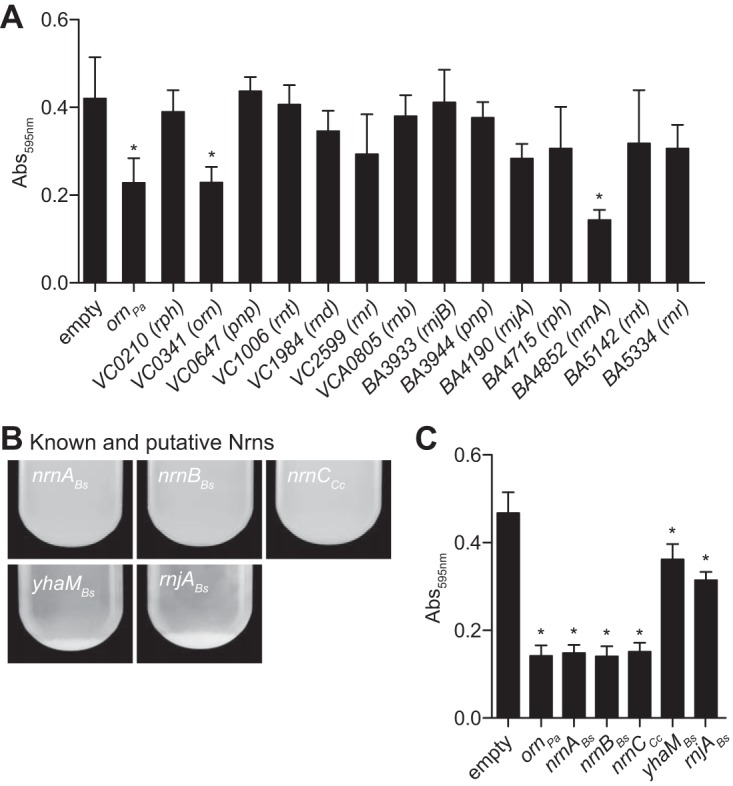
*nrnA*, *nrnB*, and *nrnC* can reduce biofilm formation and aggregation by PA14 Δ*orn*. (A) Quantification of the crystal violet assay for pellicle biofilm formation of the PA14 Δ*orn* strain with either empty vector or complementation by indicated genes carried on a pMMB-based single-copy IPTG-inducible plasmid after 24 h of static growth. (B and C) Photographs of the aggregation assay (B) and quantification of the crystal violet assay (C) for pellicle biofilm formation of PA14 Δ*orn* complemented with the indicated genes carried on a pMMB-based plasmid. Values shown are the averages and standard deviations (SD) of the results from three independent experiments. *, *P* < 0.05, Student's unpaired two-tailed *t* test.

In addition to aggregation, the Δ*orn* mutant forms more pellicle biofilm than the wild type (9). The pellicle biofilm was assayed using a crystal violet microtiter plate biofilm assay (28). Complementation of a PA14 Δ*orn* mutant with *orn_Pa_* decreased biofilm 2-fold compared to the empty vector (*P* < 0.05) (Fig. 2A). The expression of *BA4852* (*nrnA_Ba_*) and *VC0341* (*orn_Vc_*) reduced the biofilm similar to the expression level of *orn_Pa_* (*P* > 0.05), while the expression of other RNases tested had no effect in the Δ*orn* strain (Fig. 2A). Similar to the aggregation assay, only *VC0341* (*orn_Vc_*) and *BA4852* (*nrnA_Ba_*) were able to reduce the enhanced biofilm formation of PA14 Δ*orn*. These results suggest that Orn and NrnA are able to degrade pGpG in V. cholerae and B. anthracis, respectively.

Both *BA4852* (*nrnA_Ba_*) and *VC0341* (*orn_Vc_*) are 3′-to-5′ exoribonucleases with known activity against short oligoribonucleotides. NrnA from B. subtilis was originally identified from a screen that rescued the growth of an *E. coli orn* conditional mutant (14). From similar screens, other RNase genes from Bacillus subtilis and Caulobacter crescentus, namely, *nrnB*, *rnjA*, *yhaM*, and *nrnC*, were also identified that could hydrolyze short oligoribonucleotides *in vitro* (14–16). We therefore asked whether these proteins could cleave pGpG by assaying for complementation of the PA14 Δ*orn* strain. *nrnA*, *nrnB*, *rnjA*, and *yhaM* were cloned from B. subtilis 168, and *nrnC* was cloned from C. crescentus CB15 and expressed in PA14 Δ*orn*. The expression of B. subtilis *nrnA* (*nrnA_Bs_*), B. subtilis *nrnB* (*nrnB_Bs_*), and C. crescentus *nrnC* (*nrnC_Cc_*) was able to prevent aggregation of the PA14 *orn* mutant, while *yhaM* and *rnjA* were not (Fig. 2B). These strains were also assayed for pellicle biofilm formation. Complementation with *nrnA_Bs_*, *nrnB_Bs_* and *nrnC_Cc_* reduced *A*_595_ readings to 0.15 ± 0.02, 0.14 ± 0.2, and 0.15 ± 0.02, respectively, compared to the vector control *A*_595_ readings at 0.47 ± 0.05 (Fig. 2C). This reduction is similar to complementation with PA14 *orn*. The expression of B. subtilis *rnaseJ1* (*rnjA_Bs_*) and B. subtilis *yhaM* (*yhaM_Bs_*) did not prevent aggregation but resulted in a modest reduction in biofilm, with *A*_595_ readings of 0.31 ± 0.02 and 0.36 ± 0.04, respectively (Fig. 2C). Combined with the aggregation data, the expression of *rnjA_Bs_* and *yhaM_Bs_* does not efficiently complement the Δ*orn* mutant. Since pGpG accumulation causes decreased c-di-GMP turnover via feedback inhibition of the phosphodiesterase responsible for linearizing c-di-GMP, these data suggest that the genes *nrnA*, *nrnB*, and *nrnC* could degrade pGpG in species that do not have *orn*.

### Orn, NrnA, NrnB, and NrnC convert pGpG to GMP.

**FIG 3:**
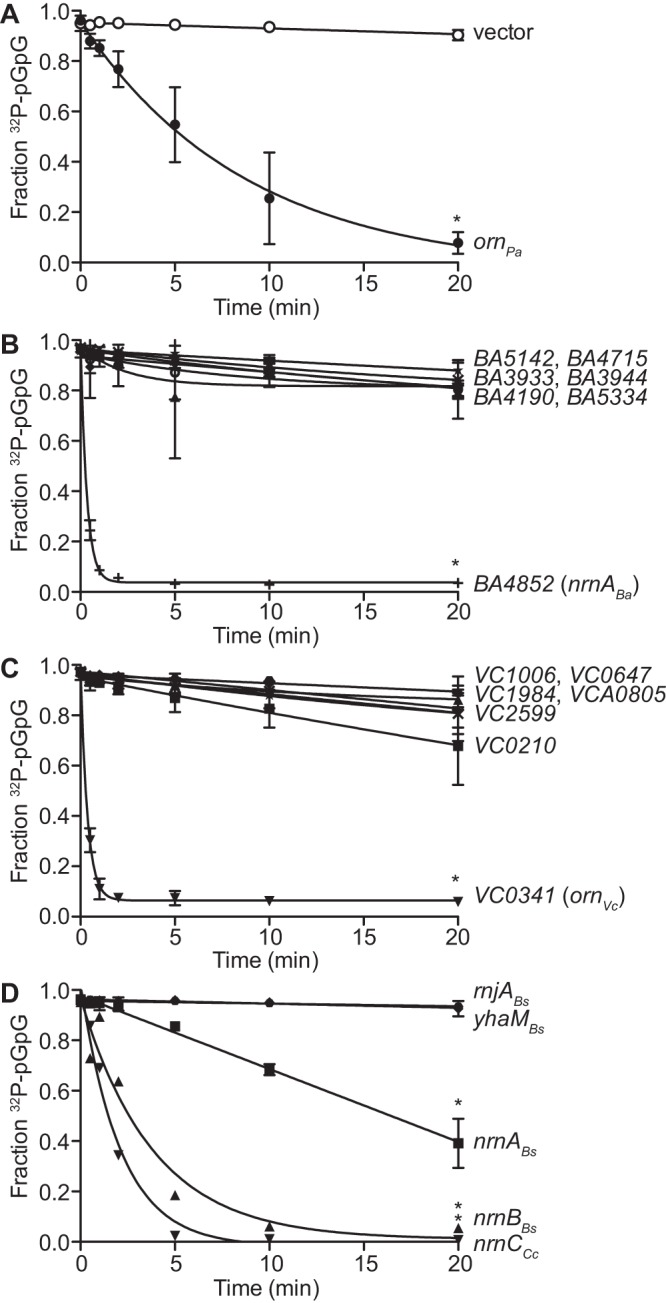
A subset of RNases can rescue PA14 Δ*orn* pGpG hydrolysis defect. The rate of pGpG cleavage by whole-cell lysates of P. aeruginosa PA14 Δ*orn* complemented with the indicated genes carried on a pMMB-based plasmid. Ten micromolar pGpG supplemented with [^32^P]pGpG tracer was monitored at the indicated times over a 20-min period. (A to C) The 3′-to-5′ exoribonucleases from B. anthracis (A), the 3′-to-5′ exoribonucleases from V. cholerae (B), and *nrnA_Bs_*, *nrnB_Bs_*, *rnjA_Bs_*,*yhaM_Bs_* and *nrnC_Cc_* (C). *, *P* < 0.05, Student's unpaired two-tailed *t* test.

The elevated c-di-GMP-related phenotypes seen in the PA14 Δ*orn* mutant strain were shown to be complemented by *orn_Pa_* but not catalytically inactive alleles of *orn_Pa_* (9, 10). As previously reported (9), the rate of pGpG turnover in whole-cell lysates was barely detectable after 20 min of incubation in the empty vector control, while plasmid-provided PA14 *orn* showed full conversion of pGpG to GMP by 20 min, with a half-life of ∼6 min (Fig. 3A). To determine the ability of each of the RNases to degrade pGpG, the lysates of PA14 Δ*orn* expressing each RNase from B. anthracis and V. cholerae were tested for their ability to hydrolyze [^32^P]pGpG to [^32^P]GMP. Of the strains expressing RNases from B. anthracis, only *BA4852* (*nrnA_Ba_*) decreased the pGpG half-life to 0.23 min (Fig. 3B). Of the strains expressing RNases from V. cholerae, *VC0341* (*orn_Vc_*) reduced the pGpG half-life to 0.25 min (Fig. 3C), while the expression of other RNases did not alter rates of pGpG degradation. When complemented with *nrnA_Bs_*, *nrnB_Bs_*, and *nrnC_Cc_*, the pGpG half-life was decreased to 16.5 min, 2.7 min, and 1.5 min, respectively (Fig. 3D). Complementation with the other RNases had pGpG hydrolysis rates similar to that of the empty vector control. These results suggest that these genes act on pGpG turnover in a manner similar to *orn* in P. aeruginosa.

**FIG 4:**
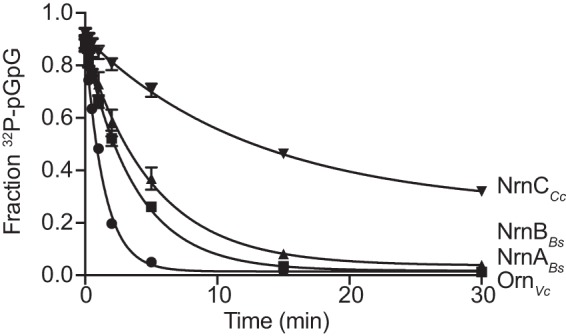
Hydrolysis of pGpG by purified RNases. The rate of [^32^P]pGpG hydrolysis by 10 nM purified Orn_*Vc*_, NrnA_*Bs*_, NrnB_*Bs*_, and NrnC_*Cc*_ incubated with 1 μM pGpG supplemented with [^32^P]pGpG tracer over a period of 30 min. Aliquots were removed and assayed by TLC. Values shown are the averages and SD of the results from three independent experiments. *, *P* < 0.05, Student's unpaired two-tailed *t* test.

To support the enzymatic activity of NrnA, NrnB, and NrnC against pGpG, purified recombinant NrnA_*Bs*_, NrnB_*Bs*_, and NrnC_*Cc*_ proteins were tested for the ability degrade pGpG. As expected, all were able to convert pGpG to GMP. When using 10 nM each enzyme, the pGpG turnover rates were determined to be 517.4 ± 7.846 nM/min for Orn_*Vc*_, 338.1 ± 14.3 nM/min for NrnA_*Bs*_, 271 ± 26.31 nM/min for NrnB_*Bs*_ and 150.6 ± 14.49 nM/min for NrnC_*Cc*_ (Fig. 4).

### HD-GYPs do not cleave pGpG in cells lacking *orn*.

**FIG 5:**
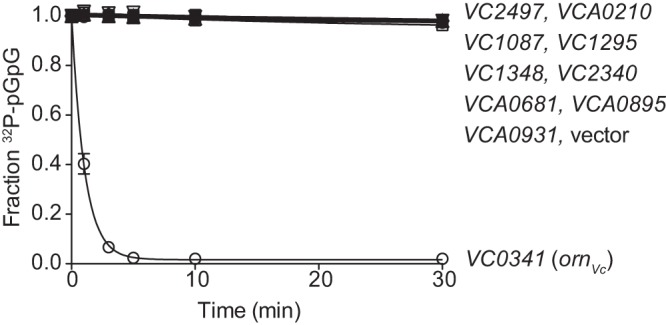
Proteins containing HD-GYP domain do not cleave pGpG in cells lacking *orn*. Lysates from PA14 Δ*orn* expressing individual genes encoding an HD-GYP domain from V. cholerae were tested for pGpG hydrolysis by monitoring the conversion of 1 µM pGpG supplemented with [^32^P]pGpG tracer to GMP. Values shown are the averages and SD of the results from three independent experiments. *, *P* < 0.05, Student's unpaired two-tailed *t* test.

Previous studies have shown that HD-GYPs can cleave both c-di-GMP and pGpG *in vitro* (8). This has led to the suggestion that proteins containing the HD-GYP domain can act to both linearize c-di-GMP and cleave pGpG *in vivo*. However, deconvolution of the *in vivo* pGpG hydrolysis activity of HD-GYP from Orn was difficult due to the essentiality of *orn* in other proteobacterial species. Using the viable P. aeruginosa Δ*orn* mutant strain, we asked whether HD-GYP proteins can cleave pGpG by expressing each of the nine genes from V. cholerae that contain a HD-GYP domain in a Δ*orn* mutant background. Lysates from these strains were tested for pGpG turnover by adding [^32^P]pGpG. Similar to the vector control, the expression of any of the HD-GYP genes failed to increase pGpG hydrolysis (Fig. 5). Since the expression of Orn_Vc_ was able to restore pGpG hydrolysis, these results indicate that HD-GYP proteins do not cleave pGpG *in vivo*.

### The intracellular concentrations of pGpG and c-di-GMP in P. aeruginosa PA14 Δ*orn* are reduced by complementation with *nrnA*, *nrnB*, *nrnC*, *VC0341*, and *BA4852*.

To confirm that these changes in phenotype were due to reducing c-di-GMP in the complementation strains, nucleotides were extracted from wild-type PA14 and the PA14 Δ*orn* strains containing empty vector or vector expressing wild-type *orn_Pa_*, *nrnA_Bs_*, *nrnB_Bs_*, *nrnC_Cc_*, *VC0341* (*orn_Vc_*), and *BA4852* (*nrnA_Ba_*), and the levels of c-di-GMP and pGpG were detected by liquid chromatography-tandem mass spectrometry (LC-MS/MS). The PA14 strain with vector control had 2.2 ± 0.4 μM pGpG, while the PA14 Δ*orn* strain with vector control had 17.4 ± 3.7 μM pGpG. Complementation of the PA14 Δ*orn* strain with all genes tested reduced pGpG and c-di-GMP levels (Table 1). Together, these results demonstrate that a specific subset of RNases can cleave pGpG to terminate c-di-GMP signaling.

**TABLE 1:** Intracellular concentration of pGpG following complementation of PA14 Δ*orn* strains

Strain	Concn (mean ± SD) (μM)^*a*^	
	pGpG	c-di-GMP
Wild-type PA14/pMMB	2.2 ± 0.4	0.016 ± 0.008
Δ*orn* mutant/pMMB	17.4 ± 3.7	0.58 ± 0.10
Δ*orn* mutant/pMMB-*orn_Pa_*	5.2 ± 1.4	0.028 ± 0.013
Δ*orn* mutant/pMMB-*orn_Vc_*	6.5 ± 1.8	0.017 ± 0.011
Δ*orn* mutant/pMMB-*nrnA_Bs_*	3.6 ± 1.4	0.022 ± 0.016
Δ*orn* mutant/pMMB-*nrnA_Bs_*	3.3 ± 1.2	0.024 ± 0.016
Δ*orn* mutant/pMMB-*nrnB_Bs_*	2.5 ± 1.1	0.023 ± 0.011
Δ*orn* mutant/pMMB-*nrnC_Cc_*	2.4 ± 1.1	0.020 ± 0.001

a From 3 experiments, calculated assuming the volume of a single bacterium equals 4.3 × 10^−1^ fl (9).

### B. subtilis 168 Δ*nrnA* Δ*nrnB* double mutant and Δ*nrnA* Δ*nrnB* Δ*yhaM* triple mutant have elevated levels of c-di-GMP.

**FIG 6:**
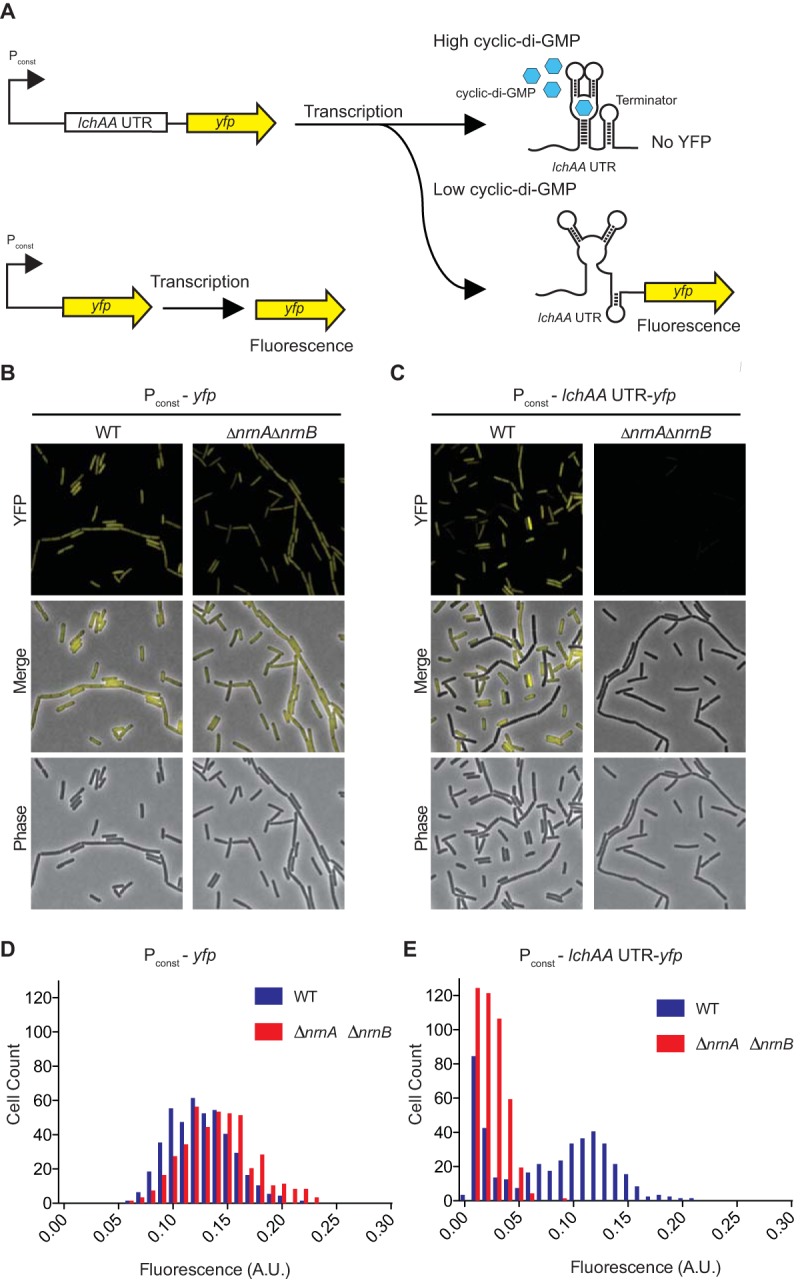
Cyclic di-GMP fluorescence riboswitch detection of c-di-GMP levels in B. subtilis 168. (A) Schematic for YFP production by P_const_-*yfp* and P_const_-*lchAA* UTR-*yfp* in response to high and low c-di-GMP. (B and C) Representative images of fluorescence of the constitutively expressed YFP reporter P_const_-*yfp* (B) or the c-di-GMP riboswitch reporter construct P_const_-*lchAA* UTR-*yfp* (C) in either B. subtilis 168 wild type (WT) or the Δ*nrnA* Δ*nrnB* double deletion mutant. (D and E) Histograms of the quantification of average fluorescence intensity of B. subtilis 168 wild type and Δ*nrnA* Δ*nrnB* with P_const_-*yfp* (D) or P_const_-*lchAA* UTR-*yfp* cells (E) (*n* = ∼300).

The ability of *nrnA_Bs_* and *nrnB_Bs_* from B. subtilis to complement the P. aeruginosa Δ*orn* mutant suggests that these enzymes could be responsible for pGpG cleavage in B. subtilis in a manner that is analogous to Orn function in P. aeruginosa. Thus, we generated an unmarked Δ*nrnA* Δ*nrnB* double mutant in B. subtilis and assayed for c-di-GMP levels using a fluorescent riboswitch reporter of c-di-GMP levels. This riboswitch reporter construct consists of a constitutively active promoter, followed by a c-di-GMP-specific riboswitch from Bacillus licheniformis found upstream of the *lch* operon (*lchAA* untranslated region [UTR]) fused to *yfp*. When the riboswitch is bound to c-di-GMP, it forms a terminator prior to *yfp*, resulting in lower fluorescence levels; when the riboswitch is not bound to c-di-GMP, it folds differently, permitting transcription elongation through the *yfp* gene, resulting in elevated fluorescence (Fig. 6A). As a control, we used a constitutively active promoter without the riboswitch before the *yfp* reporter (P_const_-*yfp*) (Fig. 6A). As expected, the control reporter showed no differences in fluorescence between the wild type and the Δ*nrnA* Δ*nrnB* double mutant, with the same histogram distribution of fluorescence intensity in the two strains (Fig. 6B and D). Inserting the *lchAA* UTR containing the c-di-GMP-specific riboswitch between the promoter and *yfp* is expected to render *yfp* expression sensitive to c-di-GMP levels. The Δ*nrnA* Δ*nrnB* mutant had very low fluorescence compared to the wild type, indicating that c-di-GMP levels are indeed higher in this strain (Fig. 6C and E). As previously reported in P. aeruginosa (9, 10), this could be due to pGpG accumulation that competitively inhibits the linearization of c-di-GMP. Although YhaM could not rescue aggregation in our assay in P. aeruginosa (Fig. 2B), it could partially reduce pellicle biofilm formation (Fig. 2C). These data, in conjunction with the report that the expression of *yhaM* could partially rescue an *E. coli orn* mutant and purified YhaM could turn over RNAs (15), led us to generate an unmarked Δ*yhaM* mutant. The Δ*yhaM* mutant had yellow fluorescent protein (YFP) levels similar to those of the parental 168 strain (Fig. S1). Furthermore, the Δ*nrnA* Δ*nrnB* Δ*yhaM* triple mutant had similar results (Fig. S2) as the Δ*nrnA* Δ*nrnB* double mutant. These results indicate that NrnA and NrnB are the enzymes primarily responsible for the degradation of pGpG in B. subtilis.

To support the changes in c-di-GMP observed with the fluorescent riboswitch reporter construct, c-di-GMP and pGpG extracted from wild-type B. subtilis and the Δ*nrnA* Δ*nrnB* mutant strains were quantified by LC-MS/MS, in which pGpG and c-di-GMP generated two daughter ions (Table 2). For the wild type, the concentration of pGpG was below the limit of detection. In contrast, the Δ*nrnA* Δ*nrnB* double-mutant strain exhibited 1.9 μM pGpG. The wild-type bacteria had 1 μM c-di-GMP, while the Δ*nrnA* Δ*nrnB* double-mutant strain had 3 μM c-di-GMP. This 3-fold increase with the double mutant agrees with the observed effect on the fluorescent c-di-GMP reporter. These results demonstrate that NrnA and NrnB degrade pGpG in B. subtilis and suggest that product inhibition of c-di-GMP linearization by pGpG is a widespread phenomenon.

**TABLE 2:** Intracellular concentrations of pGpG and c-di-GMP in B. subtilis WT and Δ*nrnA* Δ*nrnB* strains

Substance	Daughter ion	Concn (mean ± SD) (μM)^*a*^		Fold change (mutant/WT)^*b*^
		WT 168	Δ*nrnA* Δ*nrnB* mutant	
pGpG	1	ND	1.8 ± 0.6	NA
	2	ND	1.8 ± 0.4	NA
c-di-GMP	1	0.8 ± 0.2	2.4 ± 0.5	3.0
	2	0.9 ± 0.1	3.0 ± 0.9	3.3

a ND, not detected.

b NA, not applicable.

## DISCUSSION

### A subset of RNases degrade pGpG.

Only Orn, NrnA, NrnB, and NrnC can degrade pGpG. These four genes have previously been referred to as nanoRNases to describe the enzymes that can cleave “extremely short oligonucleotides” that are shorter than microRNA (14). pGpG and other linearized dinucleotides from signaling cyclic dinucleotides are two-nucleotide-long RNA molecules and represent appropriate substrates for nanoRNases. Despite being functionally similar, these four proteins contain different domains and different catalytic sites. Orn belongs to the RNase T superfamily (Pfam PF00929), NrnA and NrnB belong to the NrnA family with two adjacent DHH and DHHA1 domains (PF01368 and PF02272) (14, 15), and NrnC belongs to the RNase D superfamily (PF01612) (16). Nonetheless, these specific proteins appear to be distinct from other members of their family, since other RNases and proteins that share these domains do not appear to cleave pGpG.

Whether additional proteins that were not identified in this study can turn over pGpG remains an outstanding question. It is possible that the transgenic approach used in this screen could result in false negatives and yet-unidentified exoribonuclease families would not have been included in the candidate for screening. A more general question is, what are the total number and identity of exoribonucleases in prokaryotes? The most well-characterized exoribonucleases are in two model organisms, E. coli and B. subtilis. E. coli encodes Orn, PNPase, Rbn RNase II, Rnd, Rnr, Rph, and Rnt (21). Of the RNases found in E. coli, B. subtilis encodes only PNPase, Rph, and Rnr (22). In the past decade, a number of additional exoribonucleases have been characterized in B. subtilis, including RNase J, NrnA, NrnB, and YhaM (14, 15). Thus, there likely are additional yet-uncharacterized genes that degrade short oligonucleotides and thus can cleave pGpG and other linear dinucleotide intermediates of cyclic dinucleotide turnover. The enzymes that complement PA14 Δ*orn* were previously identified through their ability to rescue lethality in a conditional *orn* mutant in E. coli. However, while YhaM and RNase J also rescued growth of the *E. coli orn* mutant, they did not complement the biofilm phenotypes observed in PA14 Δ*orn*. These differences indicate that complementation of Orn essentiality in E. coli is a distinct phenotype from complementation of the *orn* activity in P. aeruginosa. Future experiments using the PA14 Δ*orn* strain as a surrogate host can allow identification of genes encoding enzymes from targeted organisms or from complex microbiomes.

### Enzymes that cleave linear dinucleotides are required to reduce cellular concentration of cyclic dinucleotides.

**FIG 7:**
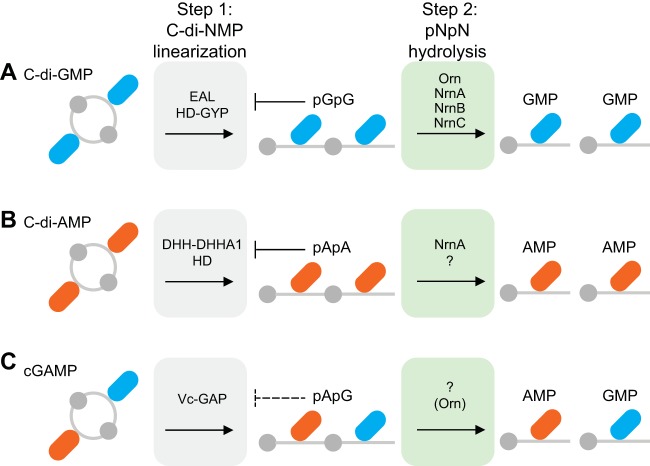
Model for degradation of cyclic dinucleotides. Cartoon of the two-step degradation process of c-di-GMP (A), c-di-AMP (B), and cGAMP (C). Step 1 is cyclic dinucleotide linearization (indicated by the gray boxes). Step 2 is dinucleotide (pNpN) hydrolysis (indicated by the green boxes). In scenarios in which the linear dinucleotide accumulates, there is feedback inhibition on the enzymes that linearize cyclic dinucleotides. Dashed lines indicate potential inhibition, and the question mark indicates the presence of additional categories of enzymes that hydrolyze dinucleotides.

The termination of cyclic dinucleotide signaling requires cleavage of the linear dinucleotide intermediate. In the absence of Orn in P. aeruginosa, pGpG is not degraded and can competitively inhibit the linearization of c-di-GMP (9, 10) (Fig. 7). As a consequence, c-di-GMP accumulates, leading to prolonged signaling and enhanced c-di-GMP-dependent phenotypes (9, 10). Data shown here for B. subtilis indicate that NrnA and NrnB degrade pGpG in this organism. YhaM is not likely to be important in pGpG turnover, since the c-di-GMP riboswitch reporter showed similar c-di-GMP levels in the parental 168 strain and the Δ*yhaM* single mutant. As observed in P. aeruginosa, the loss of the primary enzymes responsible for pGpG hydrolysis in B. subtilis leads to an accumulation of pGpG and c-di-GMP. These results suggest that feedback inhibition by pGpG on the enzymes that linearize c-di-GMP is a conserved property of c-di-GMP signaling. This feedback inhibition appears also to hold true for bis-(3′-5′)-cyclic dimeric AMP (c-di-AMP) signaling. c-di-AMP is linearized by enzymes that contain HD (29) and DHH-DHHA1 (30) domains. Recent studies of PDE2 in Staphylococcus aureus revealed that this protein cleaves 5′-*O*-phosphonoadenylyl-(3′→5′)-adenosine (pApA) in c-di-AMP signaling (31). Furthermore, in the absence of *pde2*, S. aureus cells accumulate both pApA and c-di-AMP (31) (Fig. 7). Together, these studies suggest that feedback inhibition by the linear dinucleotide product of cyclic dinucleotide turnover may be conserved. For 3′-5′ cyclic GMP-AMP (cGAMP) (32), linearization to pApG is mediated by three V-cGAP enzymes (33). How the pApG linear product of cGAMP is hydrolyzed to mononucleotide is currently unknown. Since cGAMP is produced in V. cholerae, we anticipate that Orn_Vc_ can serve to degrade both 5′-phosphoadenylyl-(3′→5′)-guanosine (pApG) and pGpG dinucleotides. Future studies with additional organisms will reveal whether feedback inhibition of linearization enzyme by linear dinucleotides is a general property of the known bacterial cyclic dinucleotide signaling molecules, c-di-GMP, c-di-AMP, and cGAMP (Fig. 7).

### Proteins containing HD-GYP domain may not cleave pGpG in cells.

Previous studies of HD-GYP proteins demonstrated that these proteins are able to degrade c-di-GMP and pGpG *in vitro* (2). *In vivo* studies in V. cholerae revealed that the expression of HD-GYP proteins reduced c-di-GMP levels (34). Furthermore, lysates of E. coli overexpressing V. cholerae HD-GYP domain proteins was able to degrade c-di-GMP into pGpG and subsequently to GMPs (35). However, the cleavage of pGpG to GMP cannot be specifically attributed to HD-GYP proteins due to the presence of Orn in the E. coli strain background. To clearly test pGpG hydrolysis activity of proteins containing an HD-GYP domain without Orn, we tested lysates of P. aeruginosa Δ*orn* expressing each of the HD-GYP genes from V. cholerae. Since the expression of these genes failed to increase pGpG cleavage, these results provide additional evidence that HD-GYP proteins do not function as the main pGpG-degrading enzymes *in vivo* (9, 10).

### NanoRNases degrade pGpG.

Unlike the linearization step of c-di-GMP, which relies on c-di-GMP-specific phosphodiesterases, our results suggest that the degradation of pGpG does not appear to require a pGpG-specific enzyme. Instead, the turnover of pGpG appears to be carried out by a subset of RNases. These RNases, dubbed nanoRNases, were identified in screens to find genes able to rescue growth in an E. coli conditional *orn* mutant and were shown to be able to turn over short oligoribonucleotides *in vitro* (14–16). However, although RNase J1 and YhaM were shown to partially rescue the *E. coli orn* growth defect (15), we did not observe that these enzymes were able to hydrolyze pGpG or rescue the P. aeruginosa *orn* biofilm and aggregation phenotypes, suggesting that not all enzymes possessing nanoRNase activity have pGpG-degrading activity. Nevertheless, the final steps of c-di-GMP and RNA turnover appear to intersect at RNases. Thus, the relative affinity for and rate of cleavage of oligoribonucleotides of different sequenced and lengths may matter during periods in which bacteria need to rapidly remove c-di-GMP. Whether this overlap in the source of oligoribonucleotides substrates for these RNases has consequences for cellular regulation or mRNA turnover is at present an open question.

The current experiments have focused on the identification of the enzymes responsible for cleaving pGpG. Since nanoRNases are hypothesized to cleave all short oligoribonucleotides regardless of sequence, we also expect them to have activity against the linearized form of the other two cyclic dinucleotide signaling molecules (pApA from c-di-AMP and pApG from cGAMP). Whether the linear pApG can also engage in product inhibition is currently unknown. However, the finding that all linear dinucleotides share the final processing enzymes that are also responsible for degrading oligoribonucleotides would not be surprising.

## MATERIALS AND METHODS

### Strains and culture conditions.

The strains, plasmids, and primers used in this study are listed in Tables S2 to S4, respectively, in the supplemental material. Bacteria were grown in LB or LB-agar supplemented with 50 μg/ml carbenicillin at 37°C, except when otherwise noted. Plasmids were induced with 1 mM isopropyl-thio-β-d-galactopyranoside (IPTG). All B. subtilis strains in this study are derived from 168. To make the Δ*yhaM*, Δ*nrnA* Δ*nrnB*, and Δ*nrnA* Δ*nrnB* Δ*yhaM* mutants, strains harboring gene knockouts of locus tags BSU29250, BSU18200, and BSU09930 were obtained from the BKE collection. The erythromycin resistance cassette inserted in each locus was then removed in each strain, and markerless deletions were created through transformation with pDR244 (36) (Bacillus Genetic Stock Center). A series of transformation protocols were performed with each BKE strain, as well as pDR244 until the double- and triple-mutant strains were achieved. Removal of the erythromycin resistance cassette was verified by Sanger sequencing. For construction of the fluorescent *yfp* reporters used in this study, integration at the *amyE* locus of 168 was performed with plasmids derived from pJG019 (GenBank accession no. KX499653.1). To construct pRSL_F4, the *lchAA* leader sequence (complete sequence is provided in the supplemental material) was synthesized (GenScript) and inserted at the HindIII restriction site of the vector. pJG019 and pRSL_F4 were transformed into 168, Δ*yhaM* mutant, Δ*nrnA* Δ*nrnB* mutant, and Δ*nrnA* Δ*nrnB* Δ*yhaM* mutant strains by using cells induced for competence through growth in nitrogen-limiting medium (37).

### Cloning.

The V. cholerae O1 biovar El Tor strain N16961 and B. anthracis Gateway-compatible ORFeome libraries were obtained from BEI Resources. The open reading frames (ORFs) were moved into the desired expression vectors (see Table S2 for primers) using the LR-Clonase II enzyme mix (Invitrogen) and introduced into chemically competent E. coli strain T7Iq cells (NEB), following the manufacturer's protocols. The *B. subtilis nrnA*, *nrnB*, *rnjA*, and *yhaM* genes and the *C. crescentus nrnC* gene were cloned using the primers shown in Table S1.

### Protein purification.

His_10_-VC0341, His_10_-BA4852, His_10_-NrnA, His_10_-NrnB, His_10_-NrnC, and His_10_-YhaM were purified from E. coli T7Iq strains containing expression plasmids (Table S2), as described previously (38). Briefly, strains were grown in LB with appropriate antibiotics at 37°C overnight, subcultured in fresh medium, and grown to an optical density at 600 nm (OD_600_) of ∼1.0, when protein production was induced with the addition of 1 mM IPTG. Induced bacteria were pelleted and resuspended in 10 mM Tris (pH 8), 100 mM NaCl, and 25 mM imidazole and frozen at −80°C until purification. Proteins were purified over a nickel-nitrilotriacetic acid (Ni-NTA) column, followed by desalting on a Sephadex G-25 column into reaction buffer. Proteins were flash-frozen in liquid nitrogen for storage at −80°C until use.

### Synthesis of radiolabeled dinucleotides.

[^32^P]pGpG was generated by the linearization of [^32^P]c-di-GMP with RocR from P. aeruginosa. For this reaction, [^32^P]c-di-GMP (0.167 μM final concentration) was incubated with RocR (20 μM final concentration) in 10 mM Tris (pH 8), 100 mM NaCl, and 5 mM MgCl_2_ at room temperature for 1 h, and the reaction was stopped by heat inactivation at 98°C for 10 min and then passed over a 3-kDa molecular weight cutoff column to remove the protein. [^32^P]c-di-GMP was enzymatically synthesized as previously described (39). Purity was checked by thin-layer chromatography (TLC).

### Cell lysate and enzymatic activity assays.

The activity of whole-cell lysates and purified proteins against [^32^P]pGpG was assayed as previously described (9). Briefly, 0.1 μM purified protein in reaction buffer (50 mM Tris [pH 8], 100 mM NaCl, and 5 mM MgCl_2_ for NrnA, NrnB, NrnC, and YhaM; 50 mM Tris [pH 8], 100 mM NaCl, and 5 mM MnCl_2_ for Orn) was incubated with the indicated concentration of pGpG spiked with 4 pM [^32^P]pGpG tracer. For cell lysates, PA14 Δ*orn* carrying the indicated complementation vectors was grown overnight, subcultured 1:100 into fresh LB supplemented with carbenicillin, induced with 100 mM IPTG, and grown at 37°C or 30°C, as indicated, to an OD_600_ of ∼0.4 with shaking. The cultures were pelleted and resuspended in a 1/10 volume of reaction buffer, adjusted to the same OD_600_, supplemented with 10 μg/ml DNase, 250 μg/ml lysozyme, and 10 mM phenylmethylsulfonyl fluoride (PMSF), and lysed by sonication. At the indicated times, aliquots were removed, and the reaction was stopped by adding an equal volume of 0.2 M EDTA (pH 8) and heated at 98°C for 10 min.

### Thin-layer chromatography.

TLC was performed as previously described (9). Briefly, 0.5 μl of each sample was spotted on polyethyleneimine-cellulose TLC plates (EMD Chemicals), dried, and developed in mobile phase consisting of 1:1.5 (vol/vol) saturated NH_4_SO_4_ and 1.5 M KH_2_PO_4_ (pH 3.60). The TLC plate was dried and imaged using the Fujifilm FLA-7000 phosphorimager (GE), and the intensity of the radiolabel was quantified using the Fujifilm Multi Gauge software version 3.0.

### Microtiter plate crystal violet biofilm assay.

For the microtiter plate crystal violet biofilm assay, overnight cultures were diluted 1:100 in LB and grown as static cultures in a 96-well polystyrene plate (Greiner) at 30°C inside a humidified chamber for 24 h. The cultures were washed of planktonic cells and stained with crystal violet, as previously described (28). The *A*_595_ was measured on a SpectraMax M5 spectrophotometer (Molecular Devices).

### Aggregation assay.

Cultures of P. aeruginosa strains were grown in 10 ml LB with the appropriate antibiotics and IPTG induction for 24 h at 37°C with shaking. Culture tubes were allowed to settle at room temperature for 30 min and were photographed.

### Fluorescence microscopy and quantification.

The B. subtilis 168 wild type (WT) and the Δ*nrnA* Δ*nrnB* and Δ*nrnA* Δ*nrnB* Δ*yhaM* mutant-derived reporter strains were grown at 37°C on LB plates supplemented with 1.5% Bacto agar and 5 μg/ml chloramphenicol, when appropriate. Single colonies were used to inoculate liquid minimal salts glycerol glutamate (MSgg) medium (40) and were grown at 37°C with shaking overnight. The following day, cultures of each strain were inoculated 1:50 on fresh medium and grown at 37°C shaking until reaching an optical density at 600 nm (OD_600_) of 1.0. Aliquots of these cultures were placed on 1.5% low-melting-point agarose MSgg pads and allowed to dry for 10 min. The agarose pads were inverted onto a glass-bottom dish (Willco Wells). Cells were imaged at room temperature using a Zeiss Axio Observer Z1 inverted fluorescence microscope, equipped with a Rolera EM-C_2_ electron-multiplying charge-coupled (EMCC) camera and an environmental chamber. Fluorescence intensity per cell was quantified using the Oufti analysis software (41). Images were analyzed and adjusted with the Fiji software (42).

### Quantification of intracellular c-di-GMP and pGpG in P. aeruginosa.

Extraction, quantification, and CFU determination were performed as previously described (9) using previously published mass spectrometry (MS) and ultraperformance liquid chromatography (UPLC) parameters (43, 44). Briefly, P. aeruginosa strains were grown overnight in LB at 37°C with shaking, subcultured 1:100 in LB, and grown at 37°C with shaking. Cells were pelleted, resuspended in 100 μl ice-cold 40:40:20 (vol/vol/vol) methanol (MeOH), acetonitrile, and water with 0.1 N formic acid, incubated 30 min at −20°C for lysis, and neutralized after a 30-min incubation with 4 μl of 15% (wt/vol) NH_4_NCO_3_. Cellular debris was pelleted, and the supernatant was removed for desiccation by a Savant SpeedVac concentrator (Thermo Scientific). Desiccated samples were suspended in 100 μl ultrapure water, and insoluble material was pelleted. The soluble supernatant was filtered through a Titan syringe filter (polyvinylidene difluoride [PVDF], 0.45 μm, 4 mm) before quantification of c-di-GMP and pGpG using LC-MS/MS on a Quattro Premier XE mass spectrometer (Waters) coupled with an Acquity ultraperformance LC system (Waters). Cyclic-di-GMP was detected in 10-μl injections of filtered extracts. For the detection of pGpG, filtered extracts were diluted 1:100 in ultrapure water, and 10-μl injections of the diluted extracts were then analyzed. The intracellular concentrations of c-di-GMP and pGpG were determined by calculating the total number of CFU in each sample and multiplying this value by the intracellular volume of a single bacterium. The total c-di-GMP and pGpG extracted in each sample were then divided by the total intracellular volume of the cells in the sample to provide the intracellular concentration of each analyte.

### Metabolite extraction and quantification of c-di-GMP and pGpG in B. subtilis.

Three independent replicates of B. subtilis 168 WT and Δ*nrnA* Δ*nrnB* mutant strains were grown overnight in liquid MSgg medium (40) with shaking at 37°C. The following day, cultures of each strain were inoculated 1:50 and grown with shaking at 37°C until reaching an optical density at 600 nm (OD_600_) of 1.0. Metabolite extraction was described previously (45). Five-milliliter cultures were passed through 0.2-μm nylon filters (EMD Millipore). Metabolism was quenched, and metabolites were extracted by inverting the filters into petri dishes that contained 1.5 ml prechilled extraction solvent composed of 40:40:20 acetonitrile-methanol-water. Dishes were placed on dry ice for 15 min before the wash was collected in microcentrifuge tubes and allowed to spin at maximum speed for 5 min at 4°C. The supernatant was then transferred to new microcentrifuge tubes and placed in a vacuum centrifuge until the metabolite extracts were dry. The detection of c-di-GMP by LC-MS/MS was described previously (46). Briefly, bacterial extract was resuspended in solvent A (10 mM tributylamine in water [pH 5.0]) and centrifuged twice to remove insoluble particles. Metabolites were then separated on a Synergi Fusion-RP column (4-μm particle size, 80-Å pore size, 150 mm by 2 mm; Phenomenex) using a Shimadzu high-performance liquid chromatography machine and simultaneously analyzed by a triple quadrupole mass spectrometer (3200 QTrap; Ab Sciex). The total run time was 20 min, at a binary flow rate of 0.5 ml · min^−1^, with 10 mM tributylamine in water (pH 5.0) as solvent A and 100% methanol as solvent B. The following gradient was performed: 0.01 min, 0% B; 4 min, 0% B; 11 min, 50% B; 13 min, 100% B; 15 min, 100% B; 16 min, 0% B; and 20 min, 0% B. c-di-GMP and pGpG were detected by multiple-reaction monitoring (MRM) under negative mode using the ion pairs 689/79 and 689/344 (c-di-GMP) and 707/79 and 707/150 (pGpG). c-di-GMP and pGpG were quantified using the Analyst software (version 1.6.2) by calculating the total peak area and were normalized by total ion current (TIC). Authentic c-di-GMP and pGpG standards were injected and analyzed alongside the samples.

## Supplementary Material

Supplemental file 1
