# Supplementary material for: A Subset of Exoribonucleases Serve as Degradative Enzymes for pGpG in c-di-GMP Signaling
Source: J Bacteriol. 2018 Nov 26;200(24):e00300-18. doi: 10.1128/JB.00300-18 (PMC6256023; doi:10.1128/JB.00300-18)
Supplement: Supplemental file 1 [file zjb999094929s1.pdf]

Supplemental information for

A subset of exoribonucleases serve as degradative enzymes for pGpG in c-di-GMP signaling

Authors & Affiliations

Mona W. Orr[a*], Cordelia A. Weiss[a], Geoffrey B. Severin[b], Husan Turdiev[a], Soo-Kyoung Kim[a], Asan Turdiev[a], Kunquing Liu[d], Benjamin P. Tu[d], Christopher M. Waters[c], Wade C. Winkler[a], Vincent T. Lee[a#]

[a] Department of Cell Biology and Molecular Genetics, University of Maryland, College Park, MD, USA

[b] Department of Biochemistry & Molecular Biology, Michigan State University, East Lansing, MI, USA

[c] Microbiology & Molecular Genetics, Michigan State University, East Lansing, MI, USA

[d] Department of Biochemistry, University of Texas Southwestern Medical Center, Dallas, TX 75390

[*]Present address: Division of Molecular and Cellular Biology, Eunice Kennedy Shriver National Institute of Child Health and Human Development, Bethesda, MD, USA

Address correspondence to Vincent T. Lee, vtlee@umd.edu

DNA sequence of P_{const}-*lchAA* leader-*yfp* reporter used for fluorescence microscopy

The promoter is underlined at the 5’ end, the *lchAA* leader sequence is bolded, and the *yfp* ORF is highlighted in yellow.

5’-GTAGCCCTTGCCTACCTAGCTTCCAAGAAAGATATCCTTACAGCACAAGAGCGG
AAAGATGTTTTGTTCTACATCCAGAACAACCTCTGCTAAAATTCCTGAAAAATTTTC
GAAAAAGTTGTTGACTTTATCTACAAGGTGTGGCATAATGTGTGTGCA**GCAGAAAA**
TGAATTTATATCAAGAAAAGCAGATAAAGGCAAACCTGCGGAAACGCAGGGACG
CAAAGCCATGGCCTAAGGTGCTGACGGTGCTACGGTTGACAGGTTGCCGAATAA
ACAGGGAGTTCGCCCGTTTTTATTCGGGCGGGCTCTTTTCTTTTTATTTCCAATAT
AATGTTTTATTGGAAACGACAAATCTGTGACAGCGTTTTTCGCTCATCGCAAAAC
CGCAACATTGCATTGCGGCTTGGCTGTTCGCATCGTCATACATAACAAGAGATAA
GCTTAAGGAGGAAAGTCACATTATGAGCAAAGGTGAAGAACTGTTCACCGGCGTT
GTGCCAATTCTGGTTGAGCTGGATGGTGACGTGAATGGCCACAAATTTTCCGTGTC
TGGTGAAGGCGAGGGTGATGCTACTTATGGCAAACTGACTCTGAAACTGATCTGTA
CCACCGGCAAACTGCCTGTTCCGTGGCCAACTCTGGTCACTACTCTGGGTTACGG
CCTGATGTGTTTTGCGCGTTACCCGGATCACATGAAACAGCATGACTTCTTCAAAT
CTGCCATGCCGGAAGGCTATGTCCAAGAACGTACGATCTTTTTCAAGGACGACGG
CAACTATAAAACCCGTGCCGAAGTTAAATTCGAGGGTGACACCCTGGTCAACCGC
ATCGAACTGAAAGGCATTGACTTCAAAGAGGACGGCAACATTCTGGGTCACAAGCT
GGAATACAACTACAACTCCCACAACGTTTACATTACTGCTGACAAGCAGAAAAACG
GCATCAAAGCAAACTTCAAGATCCGTCACAACATTGAAGATGGTGGCGTACAGCTG
GCAGATCACTACCAGCAGAACACTCCAATCGGTGATGGCCCAGTACTGCTGCCAG
ATAACCATTACCTGTCCTACCAGAGCAAACTGTCTAAAGACCCGAACGAAAAACGT
GACCACATGGTACTGCTGGAATTTGTTACCGCGGCAGGCATTACCCACGGTATGG
ACGAACTGTATAAATAAGCTAGCAAAAACCCCGCCCCTGACAGGGCGGGGTTTTTT
TT-3’

Figure S1. Cyclic di-GMP fluorescence riboswitch detection of c-di-GMP levels in *B. subtilis* 168 Δ*yhaM* deletion mutant. Representative images of fluorescence of the constitutively expressed YFP reporter P_{const}-*yfp* (A) or the c-di-GMP riboswitch reporter construct P_{const}-*lchAA* UTR-*yfp* (B) in either *B. subtilis* 168 wild type (WT) or the Δ*yhaM* mutant. Histograms of the quantification of average fluorescence intensity of *B. subtilis* 168 wild type and Δ*yhaM* with P_{const}-*yfp* (C) or P_{const}-*lchAA* UTR-*yfp* cells (D) (n ~ 300).

Figure S2. Cyclic di-GMP fluorescence riboswitch detection of c-di-GMP levels in *B. subtilis* 168 and triple deletion mutant Δ*nrnA* Δ*nrnB* Δ*yhaM*. Representative images of fluorescence of the constitutively expressed YFP reporter P_{const}-*yfp* (A) or the c-di-GMP riboswitch reporter construct P_{const}-*lchAA* UTR-*yfp* (B) in either *B. subtilis* 168 wild type (WT) or the triple deletion mutant Δ*nrnA* Δ*nrnB* Δ*yhaM*. Histograms of the quantification of average fluorescence intensity of *B. subtilis* 168 wild type and Δ*nrnA* Δ*nrnB* Δ*yhaM* with P_{const}-*yfp* (C) or P_{const}-*lchAA* UTR-*yfp* cells (D) (n ~ 300).

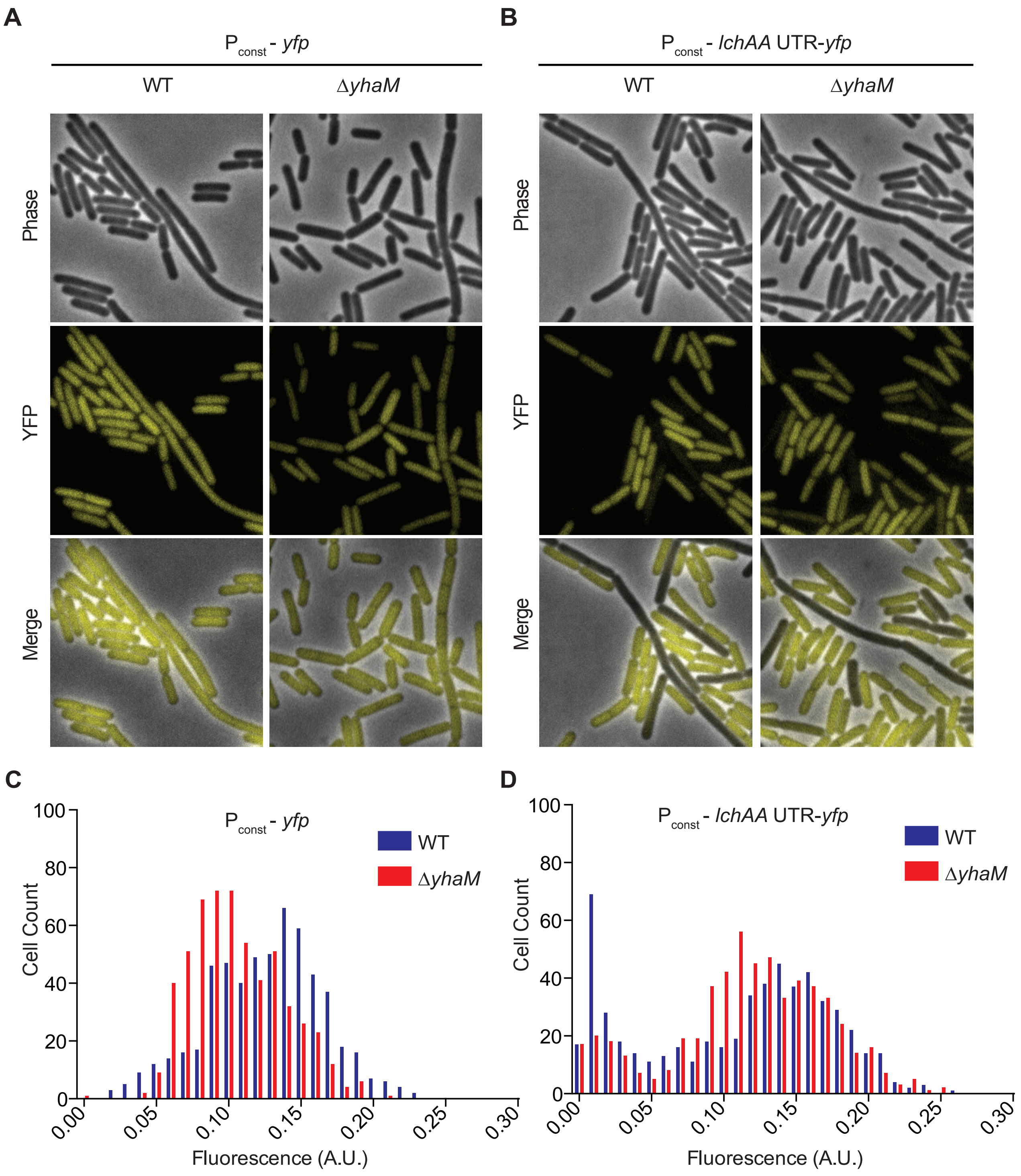

Figure S1. Cyclic di-GMP fluorescence riboswitch detection of c-di-GMP levels in *B. subtilis* 168 Δ*yhaM* deletion mutant.

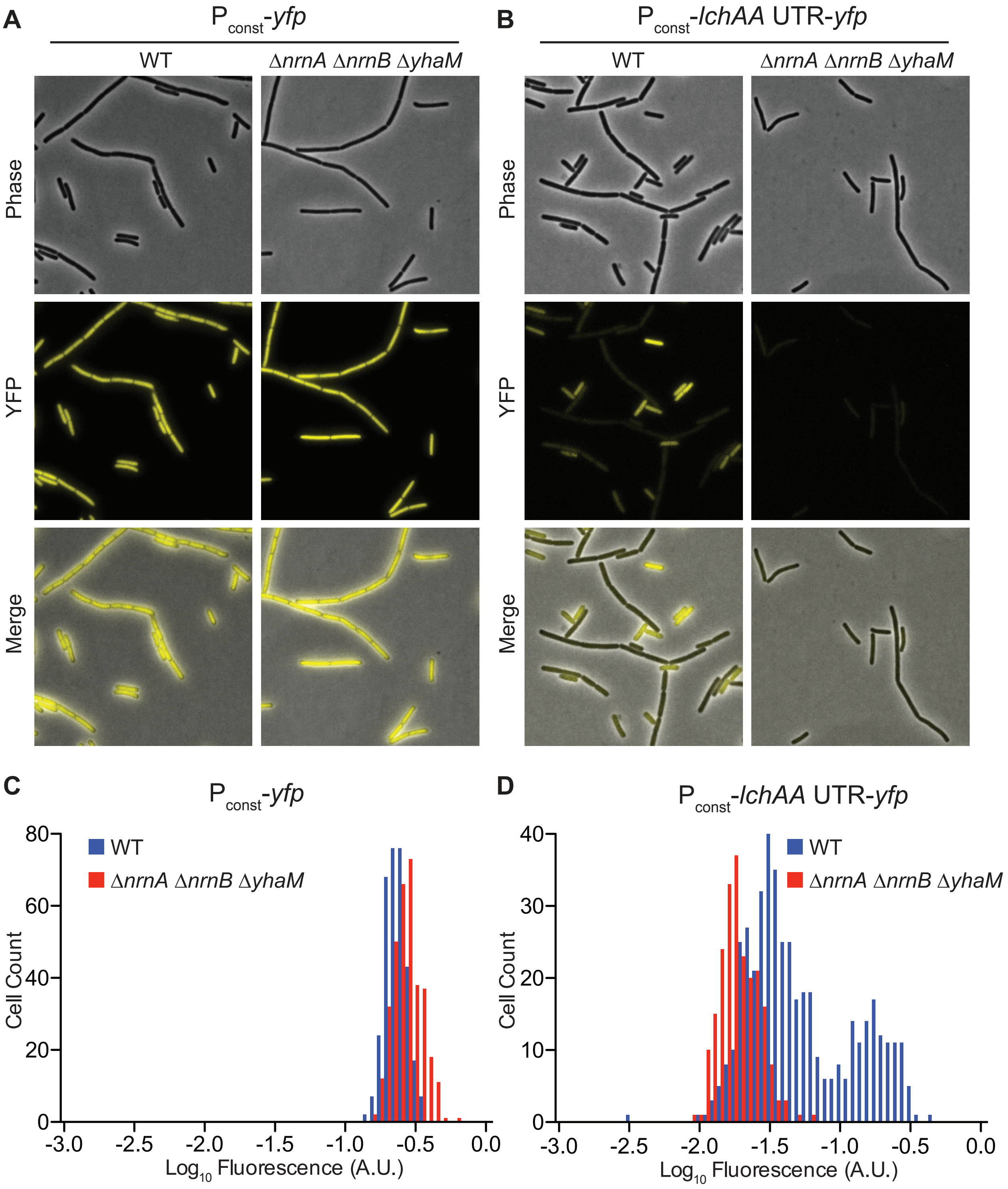

Figure S2. Cyclic di-GMP fluorescence riboswitch detection of c-di-GMP levels in *B. subtilis* 168 and triple deletion mutant Δ*nrnA* Δ*nrnB* Δ*yhaM*.

Table S1. HMMer search results for exoribonucleases Pfam domains found in *Bacillus anthracis* and *Vibrio cholerae* and other bacteria

PFAM domain	Gene number	Gene name	E-value	Score
DHH (PF01368.19)	BA2826	*ppaC*	3.40E-32	109.7
	BA4852	*nrnA*	1.40E-14	53.2
	BA5719	*gdpP*	3.70E-23	80.7
	VC1687		5.50E-22	77
	VC2417	*recJ*	1.10E-09	37.4
	NrnB	*nrnB* (*B. subtilis*)		
DHHA1 (PF02272.18)	BA3684	*alaXL*	1.10E-12	47.6
	BA4616	*alaS*	1.30E-30	105.6
	BA4639	*recJ*	1.60E-21	76.3
	VC0545	*alaS*	3.50E-30	104.3
PNPase (PF03726.13)	BA3944	*pnp*	4.30E-16	58.2
	VC0647	*pnp*	5.30E-26	90
Rnase BN (PF12706.6)	BA1088	*yhfI*	8.90E-22	76.6
	BA2247		8.70E-41	138.7
	BA2399		1.70E-07	30
	BA2405	*yobT*	2.00E-07	29.8
	BA2688	*yjfR*	2.10E-41	140.7
	BA3118		2.70E-08	32.6
	BA4275		5.00E-07	28.5
	BA4364	*rnz*	1.30E-20	72.8
	BA4485		6.70E-09	34.6
	BA4860		6.30E-25	86.9
	BA5711	*yycJ*	2.10E-20	72.2
	VC0376		8.80E-42	141.9
	VC0783		1.10E-07	30.6
	VCA0248		1.10E-11	43.7
Rnase B (PF08206.10)	BA5334	*vacB*	8.10E-32	107.7
	VC2599	*rnr*	6.00E-25	85.8
	VCA0805	*rnb*	2.10E-17	61.6
Rnase D (PF00570.22)	BA2818	*recQ2*	1.30E-23	81.8
	VC0108		4.70E-40	136.2
	VC0196	*recQ*	2.00E-23	81.2
	VC1984	*rnd*	3.00E-09	35.8
Rnase J (PF07521.11)	BA1640		1.70E-12	46.1
	BA1737		3.50E-07	29.1
	BA3933	*rnjB*	9.90E-13	46.9
	BA4190	*rnjA*	1.10E-10	40.3
	VC0264		1.50E-15	55.9
Rnase PH (PF01138.20)	BA4715	*rph*	1.30E-23	83
	VC0210	*rph*	1.70E-24	85.9
Rnase T (PF00929.23)	BA1565	*dinG*	3.80E-32	111.1
	BA3593		3.50E-21	75.5
	BA3672		5.50E-36	123.6
	BA3955	*polC*	1.60E-42	144.9
	BA5142	*kapD*	8.50E-23	80.7
	VC0299		3.00E-20	72.4
	VC0341	*orn*	8.40E-36	123
	VC1006	*rnt*	1.30E-42	145.1
	VC1234	*sbcB*	5.30E-24	84.7
	VC1290		1.90E-15	56.8
	VC1377		4.30E-12	45.9
	VC2233		5.60E-40	136.6
	NrnC	*nrnC* (*C. cresentus*)		

Table S2. Strains

Strain	Description	Source
P. aeruginosa PA14	Wild type	Rahme *et al*.
P. aeruginosa PA14 Δ*orn*	In-frame deletion of *orn*, oligoribonuclease	Orr *et al.*
P. aeruginosa PA14 Δ*orn* pMMB	*P. aeruginosa* PA14 Δ*orn* carrying pMMB	Orr *et al.*
P. aeruginosa PA14 Δ*orn* pMMB-Pa *orn*	*P. aeruginosa* PA14 Δ*orn* carrying pMMB with Pa *orn*	Orr *et al.*
P. aeruginosa PA14 Δ*orn* pMMB-Bs *nrnA*	*P. aeruginosa* PA14 Δ*orn* carrying pMMB with Bs *nrnA*	This study
P. aeruginosa PA14 Δ*orn* pMMB-Bs *nrnB*	*P. aeruginosa* PA14 Δ*orn* carrying pMMB with Bs *nrnB*	This study
P. aeruginosa PA14 Δ*orn* pMMB-Cc *nrnC*	*P. aeruginosa* PA14 Δ*orn* carrying pMMB withCc *nrnC*	This study
P. aeruginosa PA14 Δ*orn* pMMB-Bs *rnjA*	*P. aeruginosa* PA14 Δ*orn* carrying pMMB withBs *rnjA*	This study
P. aeruginosa PA14 Δ*orn* pMMB-Bs *yhaM*	*P. aeruginosa* PA14 Δ*orn* carrying pMMB with Bs *yhaM*	This study
P. aeruginosa PA14 Δ*orn* pMMB *B. anthracis BA1088 (yhfI)*	*P. aeruginosa* PA14 Δ*orn* carrying pMMB with *B. anthracis BA1088 (yhfI)*	This study
P. aeruginosa PA14 Δ*orn* pMMB *B. anthracis BA1565 (dinG)*	*P. aeruginosa* PA14 Δ*orn* carrying pMMB with *B. anthracis BA1565 (dinG)*	This study
P. aeruginosa PA14 Δ*orn* pMMB *B. anthracis BA1640*	*P. aeruginosa* PA14 Δ*orn* carrying pMMB with *B. anthracis BA1640*	This study
P. aeruginosa PA14 Δ*orn* pMMB *B. anthracis BA1737*	*P. aeruginosa* PA14 Δ*orn* carrying pMMB with *B. anthracis BA1737*	This study
P. aeruginosa PA14 Δ*orn* pMMB *B. anthracis BA2247*	*P. aeruginosa* PA14 Δ*orn* carrying pMMB with *B. anthracis BA2247*	This study
P. aeruginosa PA14 Δ*orn* pMMB *B. anthracis BA2399*	*P. aeruginosa* PA14 Δ*orn* carrying pMMB with *B. anthracis BA2399*	This study
P. aeruginosa PA14 Δ*orn* pMMB *B. anthracis BA2405 (yobT)*	*P. aeruginosa* PA14 Δ*orn* carrying pMMB with *B. anthracis BA2405 (yobT)*	This study
P. aeruginosa PA14 Δ*orn* pMMB *B. anthracis BA2688 (yjfR)*	*P. aeruginosa* PA14 Δ*orn* carrying pMMB with *B. anthracis BA2688 (yjfR)*	This study
P. aeruginosa PA14 Δ*orn* pMMB *B. anthracis BA2818 (recQ)*	*P. aeruginosa* PA14 Δ*orn* carrying pMMB with *B. anthracis BA2818 (recQ)*	This study
P. aeruginosa PA14 Δ*orn* pMMB *B. anthracis BA2826 (ppaC)*	*P. aeruginosa* PA14 Δ*orn* carrying pMMB with *B. anthracis BA2826 (ppaC)*	This study
P. aeruginosa PA14 Δ*orn* pMMB *B. anthracis BA3188*	*P. aeruginosa* PA14 Δ*orn* carrying pMMB with *B. anthracis BA3188*	This study
P. aeruginosa PA14 Δ*orn* pMMB *B. anthracis BA3593*	*P. aeruginosa* PA14 Δ*orn* carrying pMMB with *B. anthracis BA3593*	This study
P. aeruginosa PA14 Δ*orn* pMMB *B. anthracis BA3672*	*P. aeruginosa* PA14 Δ*orn* carrying pMMB with *B. anthracis BA3672*	This study
P. aeruginosa PA14 Δ*orn* pMMB *B. anthracis BA3684 (alaXL)*	*P. aeruginosa* PA14 Δ*orn* carrying pMMB with *B. anthracis BA3684 (alaXL)*	This study
P. aeruginosa PA14 Δ*orn* pMMB *B. anthracis BA3933 (rnjB)*	*P. aeruginosa* PA14 Δ*orn* carrying pMMB with *B. anthracis BA3933 (rnjB)*	This study
P. aeruginosa PA14 Δ*orn* pMMB *B. anthracis BA3944 (pnp)*	*P. aeruginosa* PA14 Δ*orn* carrying pMMB with *B. anthracis BA3944 (pnp)*	This study
P. aeruginosa PA14 Δ*orn pMMB B. anthracis BA4190 (rnjA)*	*P. aeruginosa* PA14 Δ*orn* carrying *pMMB with B. anthracis BA4190 (rnjA)*	This study
P. aeruginosa PA14 Δ*orn* pMMB *B. anthracis BA4275*	*P. aeruginosa* PA14 Δ*orn* carrying pMMB with *B. anthracis BA4275*	This study
P. aeruginosa PA14 Δ*orn* pMMB *B. anthracis BA4364 (rnz)*	*P. aeruginosa* PA14 Δ*orn* carrying pMMB with *B. anthracis BA4364 (rnz)*	This study
P. aeruginosa PA14 Δ*orn* pMMB *B. anthracis BA4485*	*P. aeruginosa* PA14 Δ*orn* carrying pMMB with *B. anthracis BA4485*	This study
P. aeruginosa PA14 Δ*orn* pMMB *B. anthracis BA4616 (alaS)*	*P. aeruginosa* PA14 Δ*orn* carrying pMMB with *B. anthracis BA4616 (alaS)*	This study
P. aeruginosa PA14 Δ*orn* pMMB *B. anthracis BA4639 (recJ)*	*P. aeruginosa* PA14 Δ*orn* carrying pMMB with *B. anthracis BA4639 (recJ)*	This study
P. aeruginosa PA14 Δ*orn* pMMB *B. anthracis BA4715 (rph)*	*P. aeruginosa* PA14 Δ*orn* carrying pMMB with *B. anthracis BA4715 (rph)*	This study
P. aeruginosa PA14 Δ*orn* pMMB *B. anthracis BA4852 (nrnA)*	*P. aeruginosa* PA14 Δ*orn* carrying pMMB with *B. anthracis BA4852 (nrnA)*	This study
P. aeruginosa PA14 Δ*orn* pMMB *B. anthracis BA4860*	*P. aeruginosa* PA14 Δ*orn* carrying pMMB with *B. anthracis BA4860*	This study
P. aeruginosa PA14 Δ*orn* pMMB *B. anthracis BA5142 (kapD)*	*P. aeruginosa* PA14 Δ*orn* carrying pMMB with *B. anthracis BA5142 (kapD)*	This study
P. aeruginosa PA14 Δ*orn* pMMB *B. anthracis BA5334 (vacR)*	*P. aeruginosa* PA14 Δ*orn* carrying pMMB with *B. anthracis BA5334 (vacR)*	This study
P. aeruginosa PA14 Δ*orn* pMMB *B. anthracis BA5711 (yycJ)*	*P. aeruginosa* PA14 Δ*orn* carrying pMMB with *B. anthracis BA5711 (yycJ)*	This study
P. aeruginosa PA14 Δ*orn* pMMB *B. anthracis BA5719 (yybT)*	*P. aeruginosa* PA14 Δ*orn* carrying pMMB with *B. anthracis BA5719 (yybT)*	This study
P. aeruginosa PA14 Δ*orn* pMMB *V. cholerae VC0108*	*P. aeruginosa* PA14 Δ*orn* carrying pMMB with *V. cholerae VC0108*	This study
P. aeruginosa PA14 Δ*orn* pMMB *V. cholerae VC0196 (recQ)*	*P. aeruginosa* PA14 Δ*orn* carrying pMMB with *V. cholerae VC0196 (recQ)*	This study
P. aeruginosa PA14 Δ*orn* pMMB *V. cholerae VC0210 (rph)*	*P. aeruginosa* PA14 Δ*orn* carrying pMMB with *V. cholerae VC0210 (rph)*	This study
P. aeruginosa PA14 Δ*orn* pMMB *V. cholerae VC0264*	*P. aeruginosa* PA14 Δ*orn* carrying pMMB with *V. cholerae VC0264*	This study

P. aeruginosa PA14 Δ*orn* pMMB *V. cholerae VC0299*	*P. aeruginosa* PA14 Δ*orn* carrying pMMB with *V. cholerae VC0299*	This study
P. aeruginosa PA14 Δ*orn* pMMB *V. cholerae VC0341 (orn)*	*P. aeruginosa* PA14 Δ*orn* carrying pMMB with *V. cholerae VC0341 (orn)*	This study
P. aeruginosa PA14 Δ*orn* pMMB *V. cholerae VC0376*	*P. aeruginosa* PA14 Δ*orn* carrying pMMB with *V. cholerae VC0376*	This study
P. aeruginosa PA14 Δ*orn* pMMB *V. cholerae VC0545 (alaS)*	*P. aeruginosa* PA14 Δ*orn* carrying pMMB with *V. cholerae VC0545 (alaS)*	This study
P. aeruginosa PA14 Δ*orn* pMMB *V. cholerae VC0647 (pnp)*	*P. aeruginosa* PA14 Δ*orn* carrying pMMB with *V. cholerae VC0647 (pnp)*	This study
P. aeruginosa PA14 Δ*orn* pMMB *V. cholerae VC0783*	*P. aeruginosa* PA14 Δ*orn* carrying pMMB with *V. cholerae VC0783*	This study
P. aeruginosa PA14 Δ*orn* pMMB *V. cholerae VC1006 (rnt)*	*P. aeruginosa* PA14 Δ*orn* carrying pMMB with *V. cholerae VC1006 (rnt)*	This study
P. aeruginosa PA14 Δ*orn* pMMB *V. cholerae VC1234 (sbcB)*	*P. aeruginosa* PA14 Δ*orn* carrying pMMB with *V. cholerae VC1234 (sbcB)*	This study
P. aeruginosa PA14 Δ*orn* pMMB *V. cholerae VC1290*	*P. aeruginosa* PA14 Δ*orn* carrying pMMB with *V. cholerae VC1290*	This study
P. aeruginosa PA14 Δ*orn* pMMB *V. cholerae VC1377*	*P. aeruginosa* PA14 Δ*orn* carrying pMMB with *V. cholerae VC1377*	This study
P. aeruginosa PA14 Δ*orn* pMMB *V. cholerae VC1687*	*P. aeruginosa* PA14 Δ*orn* carrying pMMB with *V. cholerae VC1687*	This study
P. aeruginosa PA14 Δ*orn* pMMB *V. cholerae VC1984 (rnd)*	*P. aeruginosa* PA14 Δ*orn* carrying pMMB with *V. cholerae VC1984 (rnd)*	This study
P. aeruginosa PA14 Δ*orn* pMMB *V. cholerae VC2233*	*P. aeruginosa* PA14 Δ*orn* carrying pMMB with *V. cholerae VC2233*	This study
P. aeruginosa PA14 Δ*orn* pMMB *V. cholerae VC2417 (recJ)*	*P. aeruginosa* PA14 Δ*orn* carrying pMMB with *V. cholerae VC2417 (recJ)*	This study
P. aeruginosa PA14 Δ*orn* pMMB *V. cholerae VC2599 (rnr)*	*P. aeruginosa* PA14 Δ*orn* carrying pMMB with *V. cholerae VC2599 (rnr)*	This study
P. aeruginosa PA14 Δ*orn* pMMB *V. cholerae VCA0248*	*P. aeruginosa* PA14 Δ*orn* carrying pMMB with *V. cholerae VCA0248*	This study
P. aeruginosa PA14 Δ*orn* pMMB *V. cholerae VCA0805 (rnb)*	*P. aeruginosa* PA14 Δ*orn* carrying pMMB with *V. cholerae VCA0805 (rnb)*	This study
NEB T7 Express I^q	T7 expression strain for use in protein purification	This study
NEB T7 Express I^q	NEB T7 Express I^q carrying pVL791 with *VC0341*	This study
NEB T7 Express I^q	NEB T7 Express I^q carrying pVL791 with Bs *nrnA*	This study
NEB T7 Express I^q	NEB T7 Express I^q carrying pVL791 with Bs *nrnB*	This study
NEB T7 Express I^q	NEB T7 Express I^q carrying pVL791 with Cc *nrnC*	This study
B. subtilis 168	Wild type	Bacillus Genetic Stock Center
BKE29250	*B. subtilis* 168 *nrnA*::*erm* - knockout of BSU29250 locus	Bacillus Genetic Stock Center
BKE18200	*B. subtilis* 168 *nrnB*::*erm* - knockout of BSU18200 locus	Bacillus Genetic Stock Center
BKE09930	*B. subtilis* 168 *yhaM*::*erm* - knockout of BSU09930 locus	Bacillus Genetic Stock Center
B. subtilis 168 Weiss108	Δ*nrnA* Δ*nrnB*	This study
B. subtilis 168 Weiss110	Δ*nrnA* Δ*nrnB* Δ*yhaM*	This study
B. subtilis 168 Weiss105	Δ*yhaM*	This study
B. subtilis 168 JG040	Wild type *amyE*::P_{const}-*yfp cat*	This study
B. subtilis 168 RSL_F4	Wild type *amyE*::P_{const}-*lchAA* leader-*yfp cat*	This study
B. subtilis 168 Weiss185	Δ*nrnA* Δ*nrnB amyE*::P_{const}-*yfp cat*	This study
B. subtilis 168 Weiss184	Δ*nrnA* Δ*nrnB amyE::*P_{const}-*lchAA* leader-*yfp cat*	This study
B. subtilis 168 Weiss159	Δ*nrnA* Δ*nrnB* Δ*yhaM amyE*::P_{const}-*yfp cat*	This study
B. subtilis 168 Weiss138	Δ*nrnA* Δ*nrnB* Δ*yhaM amyE*::P_{const}-*lchAA* leader-*yfp cat*	This study
B. subtilis 168 Weiss255	Δ*yhaM amyE*::P_{const}-*yfp cat*	This study
B. subtilis 168 Weiss256	Δ*yhaM amyE*::P_{const}-*lchAA* leader-*yfp cat*	This study

References

Orr *et al.* (2015) Oligoribonuclease is the primary degradative enzyme for pGpG in *Pseudomonas aeruginosa* that is required for cyclic-di-GMP turnover. *PNAS* 112(36):E5048-57.
Rahme *et al.* (1995) Common virulence factors for bacterial pathogenicity in plants and animals. *Science* 268(5219):1899-1902.

Table S3. Plasmids

Plasmid	Description	Reference
pMMB	low copy vector with tac promoter and broad host range	Fürste *et al.*
pMMB(GW)	pMMB with Gateway cassette	Wolfgang *et al*.
pMMB-Pa *orn*	pMMB with PA14 *orn*	Orr *et al.*
pMMB-Bs *nrnA*	pMMB with *B. subtilis nrnA*	This study
pMMB-Bs *nrnB*	pMMB with *B. subtilis nrnB*	This study
pMMB-Cc *nrnC*	pMMB with *C. crescentus nrnC*	This study
pMMB-Bs *rnjA*	pMMB with *B. subtilis rnjA*	This study
pMMB-Bs *yhaM*	pMMB with *B. subtilis yhaM*	This study
pMMB-*BA1088*	pMMB with *B. anthracis BA1088 (yhfI)*	This study
pMMB-*BA1565*	pMMB with *B. anthracis BA1565 (dinG)*	This study
pMMB-*BA1640*	pMMB with *B. anthracis BA1640*	This study
pMMB-*BA1737*	pMMB with *B. anthracis BA1737*	This study
pMMB-*BA2247*	pMMB with *B. anthracis BA2247*	This study
pMMB-*BA2399*	pMMB with *B. anthracis BA2399*	This study
pMMB-*BA2405*	pMMB with *B. anthracis BA2405 (yobT)*	This study
pMMB-*BA2688*	pMMB with *B. anthracis BA2688 (yjfR)*	This study
pMMB-*BA2818*	pMMB with *B. anthracis BA2818 (recQ)*	This study
pMMB-*BA2826*	pMMB with *B. anthracis BA2826 (ppaC)*	This study
pMMB-*BA3188*	pMMB with *B. anthracis BA3188*	This study
pMMB-*BA3593*	pMMB with *B. anthracis BA3593*	This study
pMMB-*BA3672*	pMMB with *B. anthracis BA3672*	This study
pMMB-*BA3684*	pMMB with *B. anthracis BA3684 (alaXL)*	This study
pMMB-*BA3933*	pMMB with *B. anthracis BA3933 (rnjB)*	This study
pMMB-*BA3944*	pMMB with *B. anthracis BA3944 (pnp)*	This study
pMMB-*BA4190*	*pMMB with B. anthracis BA4190 (rnjA)*	This study
pMMB-*BA4275*	pMMB with *B. anthracis BA4275*	This study
pMMB-*BA4364*	pMMB with *B. anthracis BA4364 (rnz)*	This study
pMMB-*BA4485*	pMMB with *B. anthracis BA4485*	This study
pMMB-*BA4616*	pMMB with *B. anthracis BA4616 (alaS)*	This study
pMMB-*BA4639*	pMMB with *B. anthracis BA4639 (recJ)*	This study
pMMB-*BA4715*	pMMB with *B. anthracis BA4715 (rph)*	This study
pMMB-*BA4852*	pMMB with *B. anthracis BA4852 (nrnA)*	This study
pMMB-*BA4860*	pMMB with *B. anthracis BA4860*	This study
pMMB-*BA5142*	pMMB with *B. anthracis BA5142 (kapD)*	This study
pMMB-*BA5334*	pMMB with *B. anthracis BA5334 (vacR)*	This study
pMMB-*BA5711*	pMMB with *B. anthracis BA5711 (yycJ)*	This study
pMMB-*BA5719*	pMMB with *B. anthracis BA5719 (yybT)*	This study
pMMB-*VC0108*	pMMB with *V. cholerae VC0108*	This study
pMMB-*VC0196*	pMMB with *V. cholerae VC0196 (recQ)*	This study
pMMB-*VC0210*	pMMB with *V. cholerae VC0210 (rph)*	This study
pMMB-*VC0264*	pMMB with *V. cholerae VC0264*	This study
pMMB-*VC0299*	pMMB with *V. cholerae VC0299*	This study
pMMB-*VC0341*	pMMB with *V. cholerae VC0341 (orn)*	This study
pMMB-*VC0376*	pMMB with *V. cholerae VC0376*	This study
pMMB-*VC0545*	pMMB with *V. cholerae VC0545 (alaS)*	This study
pMMB-*VC0647*	pMMB with *V. cholerae VC0647 (pnp)*	This study
pMMB-*VC0783*	pMMB with *V. cholerae VC0783*	This study
pMMB-*VC1006*	pMMB with *V. cholerae VC1006 (rnt)*	This study
pMMB-*VC1234*	pMMB with *V. cholerae VC1234 (sbcB)*	This study
pMMB-*VC1290*	pMMB with *V. cholerae VC1290*	This study
pMMB-*VC1377*	pMMB with *V. cholerae VC1377*	This study
pMMB-*VC1687*	pMMB with *V. cholerae VC1687*	This study
pMMB-*VC1984*	pMMB with *V. cholerae VC1984 (rnd)*	This study
pMMB-*VC2233*	pMMB with *V. cholerae VC2233*	This study
pMMB-*VC2417*	pMMB with *V. cholerae VC2417 (recJ)*	This study
pMMB-*VC2599*	pMMB with *V. cholerae VC2599 (rnr)*	This study
pMMB-*VCA0248*	pMMB with *V. cholerae VCA0248*	This study
pMMB-*VCA0805*	pMMB with *V. cholerae VCA0805 (rnb)*	This study
pMMB-VC1087	pMMB with *V. cholerae VC1087*	This study
pMMB-VC1295	pMMB with *V. cholerae VC1295*	This study
pMMB-VC1348	pMMB with *V. cholerae VC1348*	This study
pMMB-VC2340	pMMB with *V. cholerae VC2340*	This study
pMMB-VC2497	pMMB with *V. cholerae VC2497*	This study
pMMB-VCA0210	pMMB with *V. cholerae VCA0210*	This study
pMMB-VCA0681	pMMB with *V. cholerae VCA0681*	This study
pMMB-VCA0895	pMMB with *V. cholerae VCA0895*	This study
pMMB-VCA0931	pMMB with *V. cholerae VCA0931*	This study
pVL791-*VC0341*	pET19-based expression vector with *V. cholerae VC0341 (orn)*	This study
pVL791-Bs *nrnA*	pET19-based expression vector with *B. subtilis nrnA*	This study
pVL791-Bs *nrnB*	pET19-based expression vector with *B. subtilis nrnB*	This study
pVL791-Cc *nrnC*	pET19-based expression vector with *C. crescentus nrnC*	This study
pJG019 (GenBank:KX499653.1)	pDG1662-based *B. subtilis* vector with P_{const}-*yfp cat* for ectopic integration at *amyE*	Goodson *et al.*
pRSL_F4	pDG1662-based *B. subtilis* vector with P_{const}-*lchAA* leader-*yfp cat* for ectopic integration at *amyE*	Weiss *et al*.

References

Fürste *et al.* (1986) Molecular cloning of the plasmid RP4 primase region in a multi-host-range *tacP* expression vector. *Gene* 48(1):119-131.

Goodson *et al.* (2017) A Broadly Conserved Antiterminator Protein Controls a Regulon of *Bacillus amyloliquefaciens* Antibiotic Gene Clusters. *Nat. Microbiol.* 2 (17003)

Orr *et al.* (2015) Oligoribonuclease is the primary degradative enzyme for pGpG in *Pseudomonas aeruginosa* that is required for cyclic-di-GMP turnover. *PNAS* 112(36):E5048-5

Weiss *et al.* (2018) Single cell microscopy reveals that levels of cyclic di-GMP vary among *Bacillus subtilis* subpopulations. Submitted.

Wolfgang *et al*. (2003) Coordinate regulation of bacterial virulence genesby a novel adenylate cyclase-dependent signaling path-way. Dev Cell (4: 253–263.

Table S4. Primers

Primer	Purpose	Sequence
mw182	forward primer to clone *B. subtilis nrnA*	AACATATGAAAACAGAATTGATCAG
mw184	reverse primer to clone *B. subtilis nrnA*	AAGGATCCCTCGTGTTCTTTACATAATGT
mw185	forward primer to clone *B. subtilis nrnB*	AACATATGTATCATTTATATTCACATAAC
mw187	reverse primer to clone *B. subtilis nrnB*	AAGGATCCCTTGCGATGTTGATTTGC
mw209	forward primer to clone *C. crescentus nrnC*	AACATATGGCCAATTTCGTTCACGAG
mw210	reverse primer to clone *C. crescentus nrnC*	AAGGATCCGCTGTGGGCGAAGATGTCC
mw205	forward primer to clone *B. subtilis rnjA*	AACATATGAAATTTGTAAAAAATGATCAG
mw206	reverse primer to clone *B. subtilis rnjA*	AAGGATCCAACCTCCATAATGATCGGC
mw207	forward primer to clone *B. subtilis yhaM*	AACATATGGCTAAAGGGATTATGCTAC
mw208	reverse primer to clone *B. subtilis yhaM*	AAGGATCCTTTATGAAATGTCGGTTTATAAAAG
